# Supplementary material for: End Sequence Analysis Toolkit (ESAT) expands the extractable information from single-cell RNA-seq data
Source: Genome Res. 2016 Oct;26(10):1397–410. doi: 10.1101/gr.207902.116 (PMC5052061; doi:10.1101/gr.207902.116)
Supplement: Supplemental Material [file supp_gr.207902.116_Supp_Materials.tar.gz › ESAT-esat_v0.1/doc/html/dir_9be31fe7960eb9499bd01579f9da3c1a.html]

ESAT: java/broad/pda/annotation Directory Reference


ESAT


- Main Page
- Related Pages
- Packages
- Classes
- Files

All Classes Namespaces Files Functions Variables Enumerator Pages

- java
- broad
- pda
- annotation

annotation Directory Reference

Directory dependency graph for annotation:

|  |  |
| --- | --- |
| Files | |
| file | BEDFileParser.java |
|  | |
| file | GTFFileParser.java |
|  | |
| file | Locus.java |
|  | |


---

Generated on Fri Aug 15 2014 15:11:15 for ESAT by  

 1.8.7
